# Conditioned Media from Human Umbilical Cord Blood-Derived Mesenchymal Stem Cells Inhibits Melanogenesis by Promoting Proteasomal Degradation of MITF

**DOI:** 10.1371/journal.pone.0128078

**Published:** 2015-05-29

**Authors:** Eun Sung Kim, Hong Bae Jeon, Hoon Lim, Ji Hyun Shin, So Jung Park, Yoon Kyung Jo, Wonil Oh, Yoon Sun Yang, Dong-Hyung Cho, Ju-Yeon Kim

**Affiliations:** 1 Graduate School of East-West Medical Science, Kyung Hee University, Yongin-si, Gyeonggi-do, Republic of Korea; 2 Biomedical Research Institute, MEDIPOST Co., Ltd., Seongnam-si, Gyeonggi-do, Republic of Korea; Sungkyunkwan University, KOREA, REPUBLIC OF

## Abstract

Human umbilical cord blood-derived mesenchymal stem cells (hUCB-MSCs) secrete various beneficial molecules, which have anti-apoptotic activity and cell proliferation. However, the effect of hUCB-MSCs in melanogenesis is largely unclear. In this study, we show that conditioned media (CM) derived from hUCB-MSCs inhibit melanogenesis by regulating microphthalmia-associated transcription factor (MITF) expression via the ERK signalling pathway. Treatment of hUCB-MSC-CM strongly inhibited the alpha-melanocyte stimulating hormone-induced hyperpigmentation in melanoma cells as well as melanocytes. Treatment of hUCB-MSC-CM induced ERK1/2 activation in melanocytes. In addition, inhibition of ERK1/2 suppressed the anti-pigmentation activity of the hUCB-MSC-CM in melanocytes and *in vitro* artificial skin models. We also found that the expression of MITF was appreciably diminished while expression of phosphorylated MITF, which leads to its proteasomal degradation, was increased in cells treated with hUCB-MSC-CM. These results suggested that hUCB-MSC-CM significantly suppresses melanin synthesis via MITF degradation by the ERK pathway activation.

## Introduction

Melanogenesis is the process of melanin synthesis in melanocytes that determines the color of the skin, hair, and eyes. Melanin plays an important role in human skin for cosmetic appearance as well as in the protection of skin from various sources that induce damage such as ultraviolet (UV) irradiation [1]. To date, many genes involved in pigmentation have been identified. Among them, microphthalmia-associated transcription factor (MITF) is the most critical factor for the regulation of melanogenesis and melanocyte function [2]. Importantly, MITF regulates not only the gene expression of melanogenic enzymes including tyrosinase and tyrosinase-related protein1/2 (TRP1/2), but also melanocyte development and survival [3, 4]. Both tyrosinase and TRP1/2 are rate-limiting melanogenic enzymes that are involved in the regulation of melanin synthesis. Tyrosinase catalyzes the two initial steps in melanin production: the hydroxylation of tyrosine to 3,4-dihydroxyphenylalanine (DOPA) and oxidation of DOPA to dopaquinone [5]. TRP1/2 catalyzes further oxidation steps in melanogenesis. Therefore, tyrosinase inhibitors such as arbutin are regarded as skin-whitening agents [6]. The expression of MITF is regulated by transactivation by various stimuli. Cyclic AMP (cAMP) increasing agents such as alpha-melanocyte stimulating hormones (α-MSH), forskolin and isobutylmethylxanthine promote melanin synthesis by transactivation of MITF [7–9]. The binding of α-MSH to its receptor, melanocortin 1 receptor activates adenylyl cyclase, inducing cAMP production and protein kinase A activation, which eventually leads to phosphorylation of cAMP responsive element binding proteins (CREB). CREB as well as the MITF promoter are regulated by some transcription factors, which are also involved in melanocyte development [2, 4]. In addition to transcriptional control, the expression of MITF is further regulated by numerous post-translational modifications such as phosphorylation and sumoylation [10–13]. The ERK pathway, a major signaling cascade functions in many signal transductions and is involved in the regulation of MITF. Indeed, activation of the ERK pathway may increase melanogenesis. Upon c-Kit activation, ERK directly phosphorylates MITF at serine 73, which triggers proteasomal degradation via enhanced ubiquitination on lysine 201 [11]. In addition, a chemical inhibitor of the ERK pathway, PD98059, induces melanin production and tyrosinase activity [14, 15]. In contrast, the phosphorylation of MITF on serine 298, a mutation related to Waardenburg syndrome type 2 (WS2), by glycogen synthase kinase 3 enhances the binding to the tyrosinase promoter [10].

Besides environmental factors such as UV irradiation and dermatological conditions, several cytokines directly or indirectly regulate the proliferation, differentiation, and melanogenesis in epidermal melanocytes [16–18]. Recently, several reports showed that diverse effects of secreted factors from stem cells in the skin. Conditioned media (CM) derived from adipocyte stem cells showed reduction of oxidative stress and melanogenesis, and induction of wound healing in skin cells [19, 20]. However, the effect of human umbilical cord blood-derived mesenchymal stem cells (hUCB-MSCs) in melanogenesis has not been well elucidated. In this study, we evaluated the effect of CM form several different stem cell sources on melanogenesis. We found that hUCB-MSC-CM suppressed α-MSH-induced pigmentation via proteasomal degradation of MITF via ERK activation in melanocytes.

## Materials and Methods

### Cell culture

The Melan-a cell line was provided by Dr DC Bennett (St. George's Hospital Medical School, London, UK). The B16F1 cell line was purchased from American Type Culture Collection (Manassas, VA, USA). The MNT1 cell line was purchased from Korea Cell Line Bank (Seoul, Korea). Normal human epidermal melanocytes from neonatal foreskin of moderately and darkly pigmented donors were purchased from Cascade Biologics (Portland, OR, USA). Melan-a cells were maintained in RPMI 1640 medium supplemented with 10% fetal bovine serum, 1% penicillin-streptomycin (Invitrogen, Carlsbad, CA, USA) and 200 nM phorbol 12-myristate 13-acetate. B16F1 cells were maintained in Dulbecco’s modified Eagle’s medium (DMEM) supplemented with 10% fetal bovine serum and 1% penicillin-streptomycin (Invitrogen). Normal human melanocytes were maintained in M-254 medium with Human Melanocyte Growth Supplements (Cascade Biologics, Inc; Mansfield, UK). MNT1 cells were maintained in minimum essential medium (MEM) containing 10% DMEM, 20 mM HEPES (Sigma; St. Louis, MO, USA), 20% fetal bovine serum and 1% penicillin-streptomycin (Invitrogen). All cells were maintained with 5% CO_2_ in a humidified chamber at 37°C.

### Culture of hUCB-MSC and preparation conditioned media from MSC

This study was approved by the Institutional Review Board of MEDIPOST Co., Ltd [21, 22]. Neonatal hUCB was collected from umbilical veins, with informed maternal consent. Mononuclear cells were isolated from hUCB by centrifugation on a Ficoll-Hypaque gradient (density: 1.077 g/cm^3^; Sigma, St. Louis, MO). Separated mononuclear cells were washed, suspended in a-minimum essential medium (α-MEM; Gibco, Carlsbad, CA) supplemented with 10% (v/v) fetal bovine serum (FBS; Gibco) and 50 mg/mL gentamicin (Gibco), and seeded at a concentration of 5 3 105 cells per centimeter square in culture flasks. Cultures were maintained at 37°C in a humidified 5% CO_2_ atmosphere with a twice-weekly medium change. After 1–3 weeks, when the monolayer of fibroblast-like adherent cell colonies had reached 80% confluence, the cells were detached with TrypLE Express (Gibco), washed, re-suspended in culture medium (a-MEM supplemented with 10% FBS and 50 mg/ml gentamicin), and subcultured. In all experiments, hUCB-MSCs used were at passage 6 [21, 22]. Then, the hUCB-MSCs derived CM was harvested after 48 hr. This collected soup was defined as hUCB-MSC-CM (hUCB-MSCs derived conditioned media). The hUCB-MSC-CM was prepared by filtration through a centrifugal filter unit (Millipore Billerica, MA, USA) and stored at 4°C or −80°C before being used for subsequent experiments.

### Reagents

α-MSH, forskolin, resveratrol (RSV), and arbutin were purchased from Sigma (St. Louis, MO, USA). PD98059 was purchased from Calbiochem (La Jolla, CA, USA). Three-dimensional human skin substitutes (Melano Derm Tissue) were purchased from MatTek (Ashland, MA, USA). The EGFP-fused MITF cDNA was purchased from Addgene (Cambridge, MA, USA).

### Measurement of tyrosinase activity

Melan-a cells were plated in six-well cell culture plate. After 24 hr, the cells were treated with hUCB-MSC-CM and resveratrol in the presence or absence of α-MSH. After 24 hr, the cells were harvested by trypsinization and washed with cold PBS. The cells were further mixed with 0.1 M phosphate buffer (pH 6.8) and left on ice for 1 hr to release tyrosinase from the melanosome, and then the cells were centrifuged at 13,000 rpm for 20 min at 4°C. The supernatants were mixed and inverted with 0.2% of L-DOPA solution. After incubating the mixtures in 37°C incubator for 30 min, tyrosinase activity was measured at 490 nm with ELISA reader (Victor X3, Perkin Elmer).

### Determination of melanin content

Melan-a, B16F1, MNT-1 and NHEM treated with indicated reagents were collected. And simply, the cellular melanin was extracted by using melanin extraction buffer (1N NaOH containing 10% DMSO) at 100°C for 30 min. Then the cellular melanin contents were determined by measuring the absorbance at 415 nm using an ELISA plate reader (Victor X3, Perkin-Elmer).

### *In vitro* 3D artificial skin model (MelanoDerm Tissue Model)

Both underdeveloped MelanoDerm tissue (MEK-300-B) and the maintenance medium for MEK-300-B (EPI-100-LLMM) were purchased from MatTek Corporation (Ashland, MA, USA). To measure melanin content, MelanoDerm tissue was incubated in 6 well plates containing the pre-warmed maintenance medium according to recommended protocols. MelanoDerm tissues were treated with control media of hUCB-MCS-CM in the presence or absence of PD98059. The medium was changed every other day with same CM for 18–20 days.

### Western blotting analysis

All cell lysates were boiled and prepared with protein sample buffer (62.5 mM Tris-HCl, pH 6.8, 25% glycerol, 2% SDS, 5% β-mercaptoethanol, 0.01% Bromophenol blue) (BioRad, Hercules, CA, USA). Then the proteins were extracted. And the proteins were separated by SDS-PAGE and transferred to PVDF membrane (BioRad). After blocking, the membranes were incubated over-night with specific primary antibodies at 4°C. Anti-Tyrosinase and anti-TRP1 antibodies were donated by V.J. Hearing (NIH, Bethesda, MD, USA). Anti-MITF (sc-5625) and anti-GFP (FL) (sc-8334) antibodies were purchased from Santa Cruz Biotechnology (Santa Cruz, CA, USA); Anti-phospho-MITF (LS-C199259) antibody was purchased from Lifespan Bioscience (Seattle, WA, USA); Anti-Phospho-p44/42 MAP kinase (p-ERK 1/2) (9101), anti-p44/42 MAP kinase (ERK 1/2) (9102), anti-Phospho-p38 MAP kinase (Thr180/Tyr182) (p-p38) (9211), anti-p38 MAP kinase (p38) (9212), anti-Phospho-SAPK/JNK (Thr183/Tyr185) (p-JNK 1/2) (9251) and anti-SAPK/JNK (JNK 1/2) (9252) antibodies were purchased from Cell Signaling Technology (Beverly, MA, USA); Anti-Actin (MAB1501) antibody was purchased from Millipore (Temecula, CA, USA). For protein detection, the membranes were incubated with HRP-conjugated secondary antibodies (Pierce, Rockford, IL, USA).

### Statistical analysis

Presented bar data shows standard error of the mean (S.E.M.). Significant differences among groups were determined using the Student’s *t*-test. Statistical significance was defined as a *p-*value <0.05

## Results

### hUCB-MSC-CM inhibits pigmentation in melanocytes and melanoma cells

**Fig 1:**
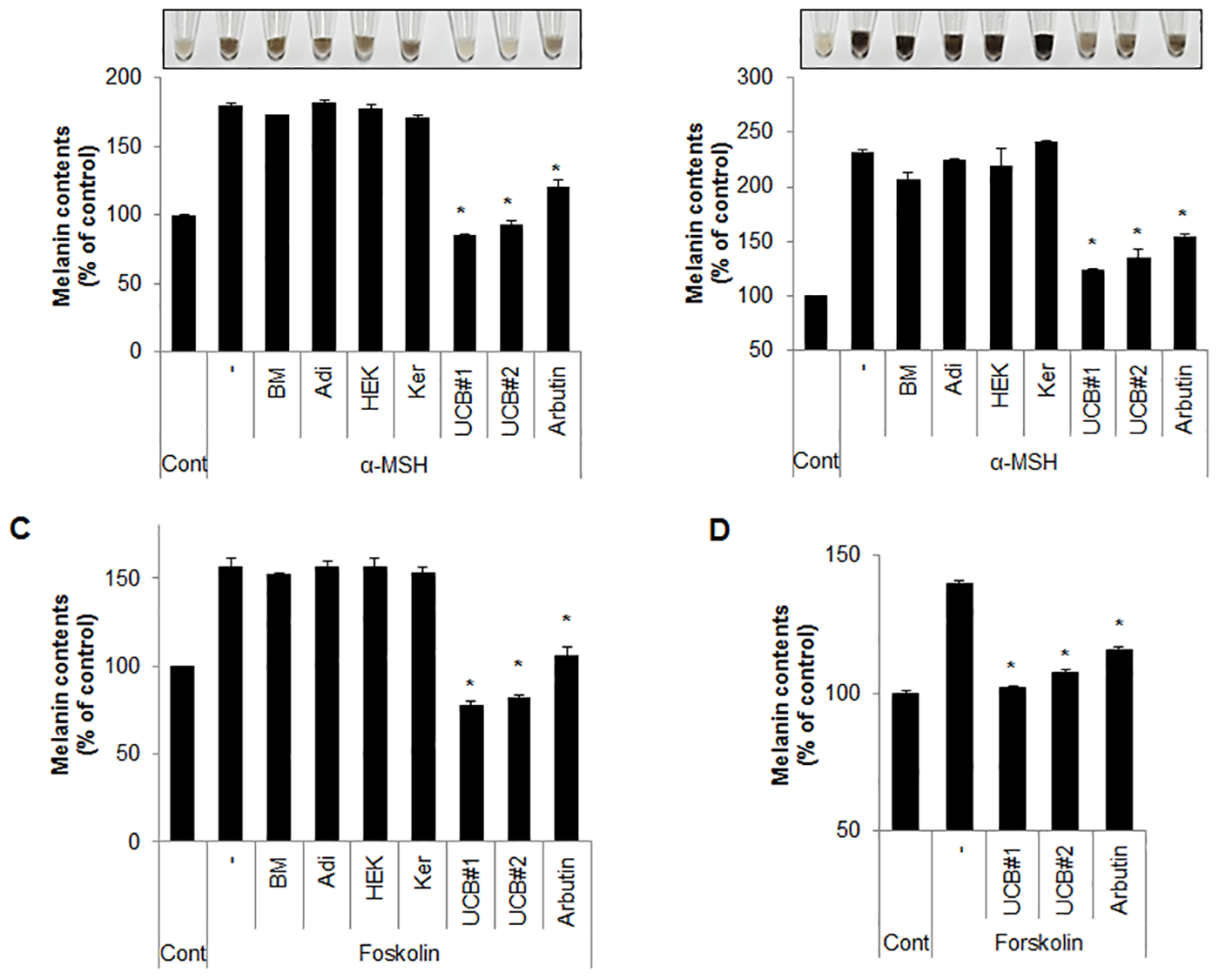
hUCB-MSC-CM suppresses α-MSH-induced melanogenesis. (A) Melan-a cells pre-treated with α-MSH (1 μM) for 24 hr were incubated with BM (medium form bone marrow mesenchymal stem cells), Adi (medium from adipocyte mesenchymal stem cells), Ker (medium from human epidermal keratinocytes), and UCB (medium from human umbilical cord blood-derived mesenchymal stem cells) for additional 24 hr. Cells were treated with arbutin (500 μM) as an anti-melanogenic agent. Then, the cells were collected (upper image) to measure cellular melanin contents (lower graph) as described in the Material and Methods section. (B) B16F1 cells pre-treated with α-MSH (1 μM) for 24 hr were incubated with BM, Adi, Ker, UBC, and arbutin (500 μM) for 24 hr. Then, the cells were collected (upper image) to examine the cellular melanin contents (lower graph). (C) Melan-a mouse melanocytes pre-treated with forskolin (20 μM) for 24 hr were incubated with BM (medium from bone marrow mesenchymal stem cells), Adi (medium from adipocyte mesenchymal stem cells), Ker (medium form human epidermal keratinocytes), or UCB (medium from human umbilical cord blood-derived mesenchymal stem cells) for additional 24 hr. Cells were treated with arbutin (500 μM) as a positive control. Then, the cells were collected to measure cellular melanin contents. (D) Normal human epidermal melanocytes were pre-treated with forskolin (20 μM) for 48 hr. The cells were further incubated with hUCB-MSC-CM or arbutin (500 μM) for 48 hr, and cellular melanin contents were measured. Data represent ± standard error of the mean (S.E.M.) from three independent experiments (n = 3,* *p*<0.02).

**Fig 2:**
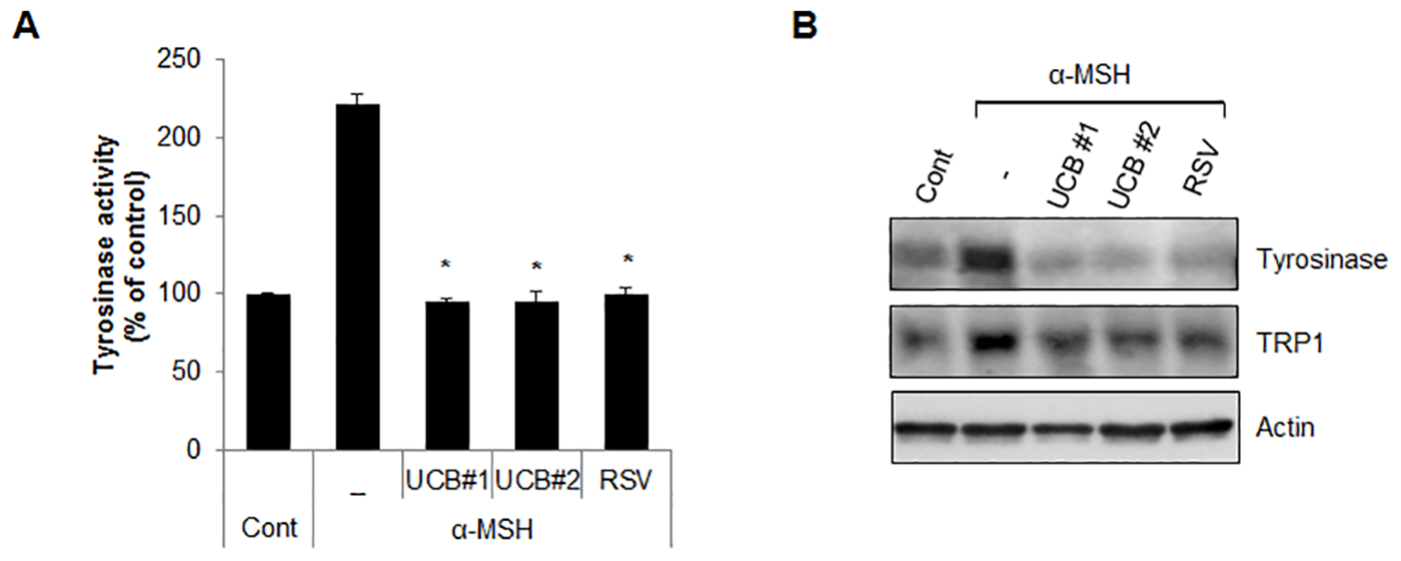
hUCB-MSC-CM inhibits tyrosinase activity and down-regulates melanogenic enzymes in melanocytes. (A, B) Melan-a cells pre-treated with α-MSH (1 μM) were incubated with conditioned medium derived from hUCB-MSCs (UCB #1, #2) or treated resveratrol (RSV) (100 μM) for 24 hr. Then, the cells were harvested to measure the tyrosinase activity (A). Expressional level of tyrosinase and TRP1 proteins was analyzed by Western blotting (B). Data represent ± standard error of the mean (S.E.M.) from more than three independent experiments, n = 3,* *p*<0.02).

**Fig 3:**
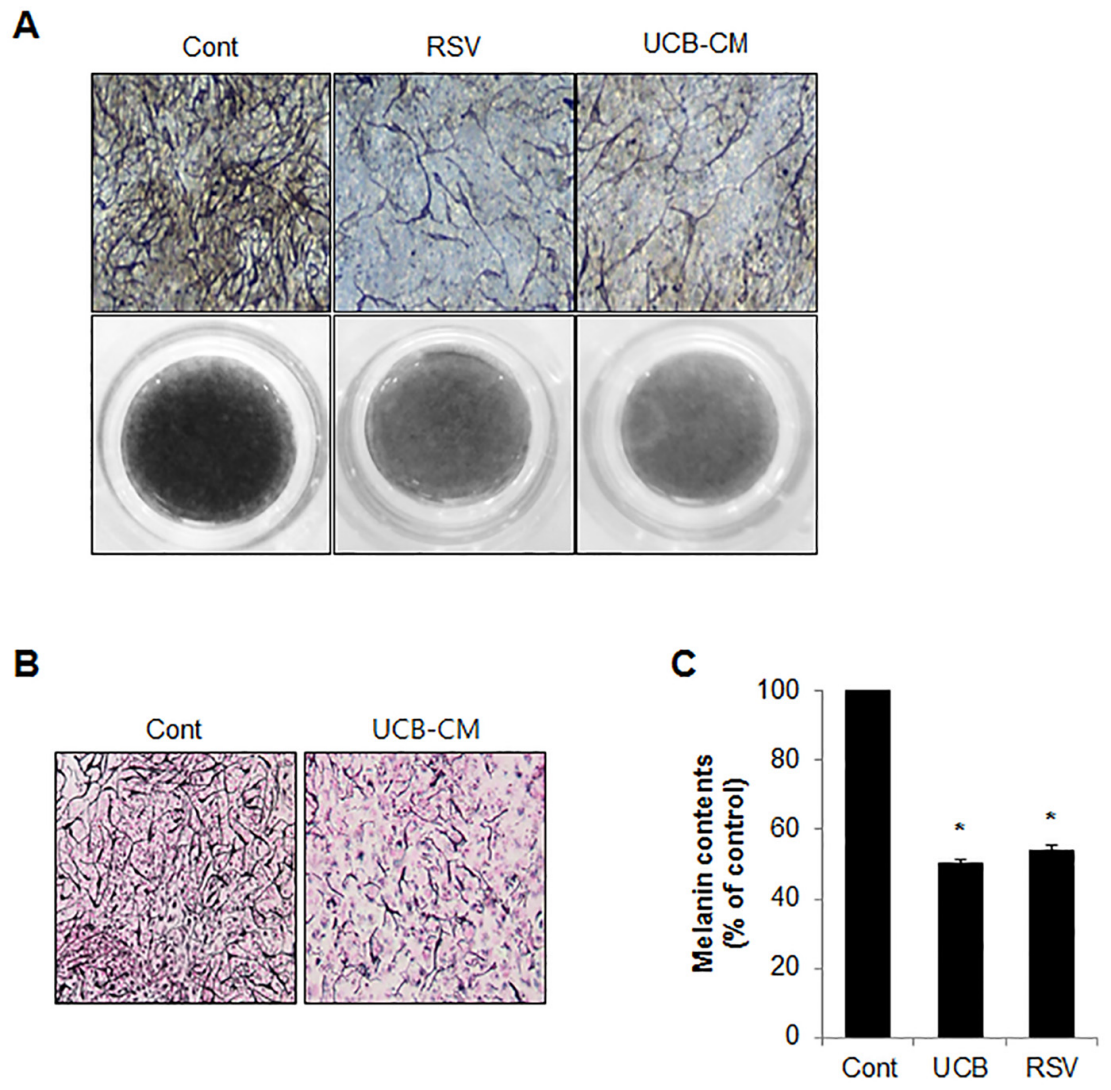
hUCB-MSC-CM suppresses melanogenesis in an artificial human skin model. (A-C) MelanoDerm tissues were incubated with either growth medium (Cont) or hUCB-MSC-CM for 20 days or resveratrol (RSV) for 18 days. Then the Melanoderm tissues were observed under a microscope (A) or analyzed by Masson-Fontana staining (B). The tissues were collected to determine the total melanin content (C). RSV was used as a positive control agent. Data represent standard error of the mean (S.E.M) from three independent experiments (n = 3, p<0.05)

hUCB-MSC-CM shows several advantages for cell differentiation potential and immune-modulation activity [23, 24]. However, the role of hUCB-MSC-CM in skin pigmentation has not been well elucidated. To address the effect of hUCB-MSC-CM on melanogenesis, both Melan-a melanocytes and B16F1 melanoma cells were stimulated with α-MSH, and the cells were further treated with CM derived from bone marrow-MSCs (BM-MSCs), adipocyte-MSCs, human epidermal keratinocytes-MSCs (HEK-MSCs) as well as hUCB-MSCs. Interestingly, cells incubated with hUCB-MSC-CM more efficiently suppressed the α-MSH-induced pigmentation than that of arbiun, a strong tyrosinase inhibitor in melanocytes and melanoma cells (Fig 1A and 1B). On the other hand, BM-MSC-CM, adipocyte-MSC-CM, and HEK-MSC-CM did not significantly influence melanin production (Fig 1A and 1B). To further confirm the anti-melanogenic effect of hUCB-MSC-CM, we employed forskolin, another well-known melanogenesis inducer in Melan-a cells and normal human epidermal melanocytes (NHEM). In accordance with previous results, the over produced melanin by forskolin was only significantly suppressed by hUCB-MSC-CM but not by other MSCs-derived CM in Melan-a cells (Fig 1C). In addition hUCB-MSC-CM also effectively inhibited forskolin-mediated pigmentation in NHEM (Fig 1D). Melanogenesis is regulated by several melanogenic proteins, including tyrosinase and TRP1/2. Thus, many anti-melanogenic agents regulate the activity or expression of melanogenic proteins. To investigate the effect of hUCB-MSC-CM on melanogenesis, tyrosinase activity was examined in Melan-a cells stimulated with α-MSH after incubating with hUCB-MSC-CM or resveratrol (RSV), a potent tyrosinase inhibitor. Treatment of hUCB-MSC-CM significantly reduced the α-MSH-stimulated tyrosinase activity in melanocytes to the same extent as RSV (Fig 2A). Furthermore, the increased expression of melanogenic enzymes such as tyrosinase and TRP1 was also reduced in hUCB-MSC-CM-treated melanocytes (Fig 2B). We additionally assessed the effect of hUCB-MSC-CM on melanogenesis in a human artificial skin model. An artificial skin sample was treated with either hUCB-MSC-CM or RSV for 13 days. Consistently, hUCB-MSC-CM efficiently suppressed pigmentation to the same extent as RSV in the human artificial skin model (Fig 3A–3C). Taken together, these results suggested that hUCB-MSC-CM negatively regulates melanogenesis in melanocytes.

### ERK1/2 activation is involved in hUCB-MSC-CM-mediated anti-melanogenesis activity

**Fig 4:**
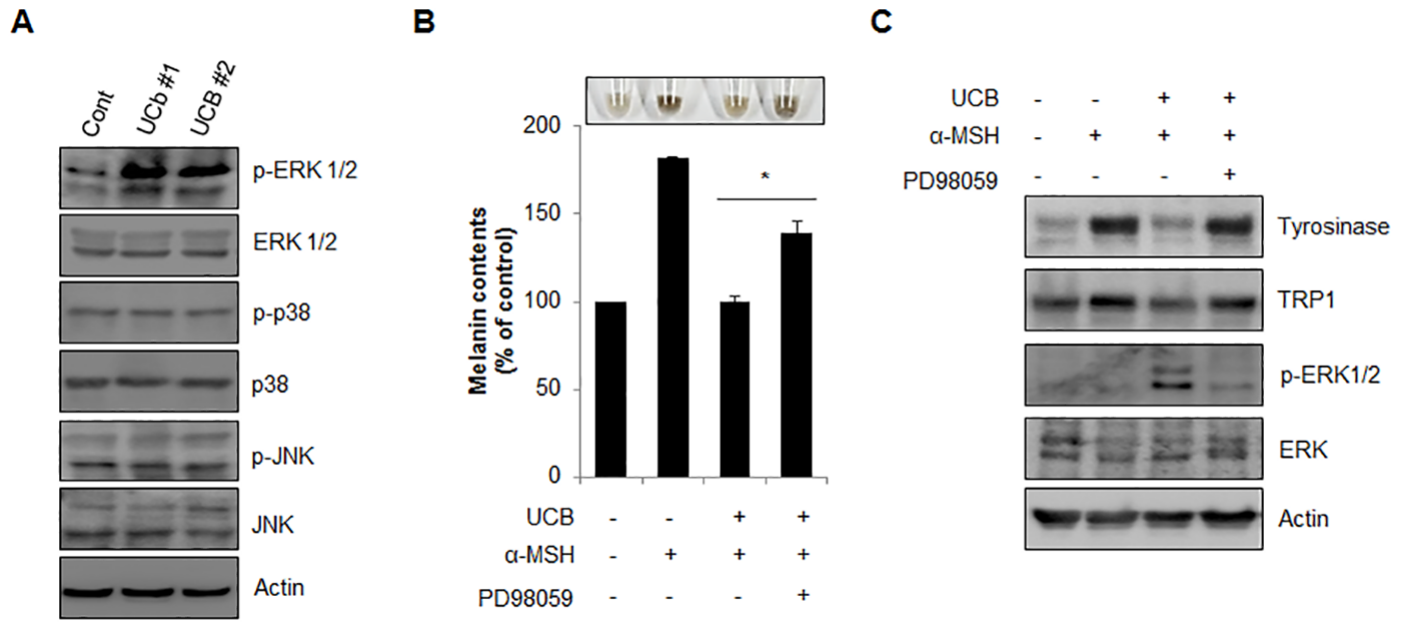
ERK1/2 activation mediates the anti-melanogenic activity of hUCB-MSC-CM in melanocytes. (A) Melan-a cells were incubated with hUCB-MSC-CM (UCB#1, #2) for 24 hr and analyzed protein expression by Western blotting with indicated antibodies. (B, C) Melan-a cells pre-treated with α-MSH (1 μM) were incubated with hUCB-MSC-CM in the presence or absence of PD98059 (20 μM) for 24 hr. Then, the cells were harvested (upper image) to measure the cellular melanin contents (B). The protein expressional level of tyrosinase, TRP1, p-ERK 1/2 and ERK 1/2 in the cells was further analyzed by Western blotting (C). The data were obtained from least three independent experiments and values are presented as the means ± S.E.M. (n>3, * *p*<0.05).

**Fig 5:**
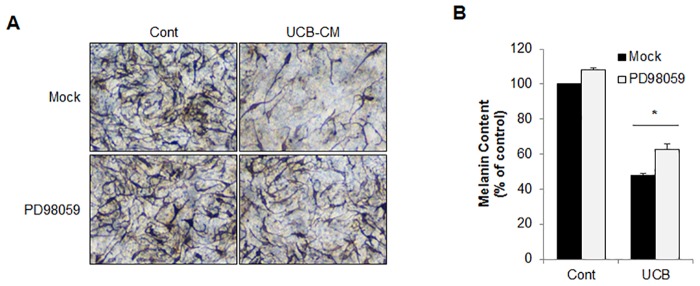
Inhibition of ERK1/2 suppressed anti-melanogenic activity of hUCB-MSC-CM in artificial human skin model. (A, B) The MelanoDerm tissues were treated with hUCB-MSC-CM in the presence or absence of PD98059 for 18 days. Then, the image was captured (A). And the tissues were collected to measure total melanin contents (B). The data were obtained from least three independent experiments and values are presented as the means ± S.E.M. (n>3, * *p*<0.05).

hUCB-MSC-CM activates a number of cellular signal transduction pathways [21, 25, 26] and melanogenesis is regulated by signaling proteins such as Wnt, ATK, and MAPK [27–29]. To explore the molecular mechanism underlying hUCB-MSC-CM-mediated anti-melanogenesis activity, we examined several kinase proteins that are involved in the regulation of melanogenesis. As shown in Fig 4A, incubation of hUCB-MSC-CM in Malan-a cells highly induced ERK1/2 phosphorylation but did not activate JNK and p38 (Fig 4A). Therefore, we further assessed the effect of ERK1/2 phosphorylation on melanogenesis in hUCB-MSC-CM-treated cells. In α-MSH-stimulated melanocytes, the reduction of melanin contents by hUCB-MSC-CM was conspicuously recovered by treatment of PD98059, an ERK1/2 inhibitor (Fig 4B). Additionally, inhibition of ERK1/2 reinstated the reduced expression of tyrosinase and TRP1 expression in hUCB-MSC-CM-treated melanocytes (Fig 4C). We further evaluated the role of ERK1/2 in melanogenesis using a human artificial skin model. In accordance, inhibition of ERK1/2 by PD98059 significantly suppressed the anti-pigmentation activity of hUCB-MSC-CM in an artificial skin model (Fig 5A and 5B). Collectively, our results suggested that the ERK pathway mediates the anti-melanogenic activity of hUCB-MSC-CM in hyper-pigmented cells.

### hUCB-MSC-CM promotes proteasomal degradation of MITF in melanocytes

**Fig 6:**
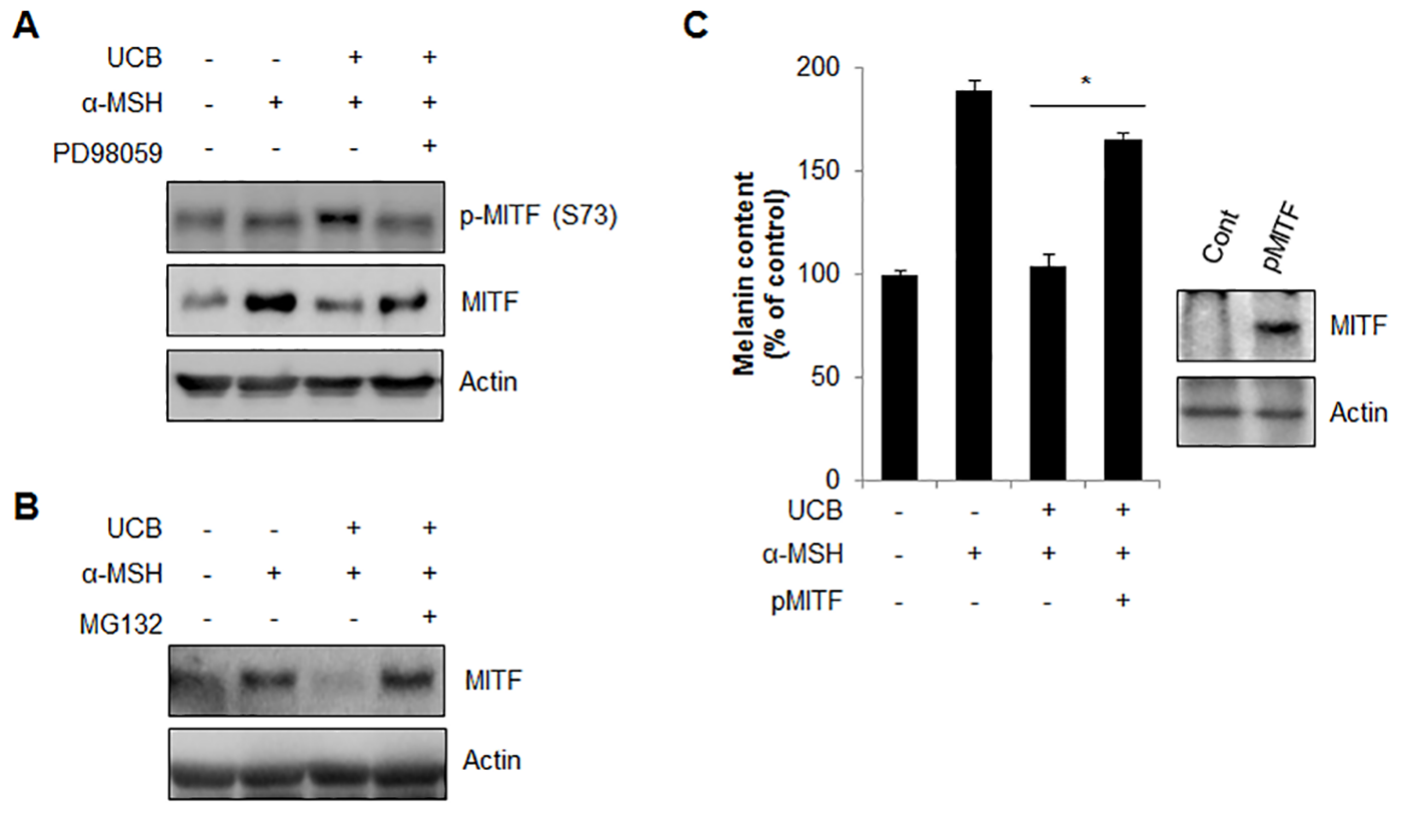
hUCB-MSC-CM induces proteasomal degradation of MITF mediated by ERK phosphorylation. (A) Melan-a cells pre-treated with α-MSH (200 nM) for 24 hr were further incubated with hUCB-MSC-CM (UCB) in the presence or absence of PD98059 (20 μM) for 24 hr. Then, the expression of phosphorylated MITF at Serine 73 and total MITF protein was examined with Western blot analysis. (B) Melan-a cells pre-treated with α-MSH (200 nM) were incubated with UCB with or without MG132 (5 μM) for an additional 24 hr. Then the MITF level was examined by Western blot analysis. (C) Human MNT-1 melanoma cells transiently transfected with pEGFP (pCont) or pEGFP-MITF (pMITF) were treated with α-MSH (1 μM). After 24 hr, the cells were further incubated with UCB for additional 24 hr. Then the cells were harvested to measure the cellular melanin contents. The over-expression of MITF was confirmed by Western blotting with anti-GFP antibody. Data represent ± standard error of the mean (S.E.M.) from more than three independent experiments, n = 3,* *p*<0.05).

It was previously reported that ERK directly phosphorylates MITF on serine 73, which leads to its proteasomal degradation [30]. Since hUCB-MSC-CM induced ERK activation and MITF is a key factor for the regulation of melanogenic enzymes, we next investigated the expression of MITF and phospho-MITF (Ser 73) in hUCB-MSC-CM-incubated cells. The expression of MITF was appreciably diminished, while phospho-MITF (Ser 73) was increased following hUCB-MSC-CM incubation in α-MSH-treated cells (Fig 6A). The reduced MITF level was restored by treating cells with a proteasome inhibitor (MG132) (Fig 6B), suggesting that hUCB-MSC-CM regulates pigmentation by modulating MITF degradation. We then examined melanin contents after overexpression of MITF in hUCB-MSC-CM-incubated cells. Ectopic expression of MITF recovered the hUCB-MSC-CM-reduced melanin contents in α-MSH-treated MNT-1 cells, as predicted (Fig 6C). Taken together, these results suggested that hUCB-MSC-CM regulates melanogenesis through the proteasomal degradation of MITF by ERK activation.

## Discussion

MSCs are multipotent stromal cells that have a self-renewal capacity, and can be differentiated into a variety of cell types such as osteoblasts, chondrocytes, myocytes and adipocytes [31, 32]. MSCs can be isolated from several different sources such as embryonic tissue, bone marrow, adipose tissue, and the placenta [33]. Among them, hUCB has recently been regarded as an alternative stem cell source for the isolation and collection of MSCs [34, 35]. MSCs have been proposed as a drug factory in many diseases because they secrete various therapeutic proteins to pathological sites [21, 34]. As a paracrine action, the secreted molecules from MSCs actively participate in anti-apoptosis, stimulation of proliferation, and removal of toxic proteins [33, 35]. Previously, we showed that CM of hUCB-MSCs contained various beneficial proteins such as growth factors and cytokines [21, 22]. In this study, we further found that CM from hUCB-MSCs efficiently inhibited α-MSH-induced hyperpigmentation by reducing MITF activity in normal melanocytes and melanoma cell lines. However, CM derived other MSCs did not show a significant effect on melanogenesis (Fig 1).

Since hUCB-MSCs are isolated from neonatal tissues, they are more primitive MSCs compared to other sources from adult, such as bone marrow, adipocytes. hUCB-MSCs have more paracrine factors than that of other more aged sources [36]. Regarding with these primitive features, hUCB-MSCs showed delayed aging processes during long term cultured condition [36]. Particular paracrine factors, such as senescence associated secretory phenotype (SASP) are much lower in hUCB-MSCs compared to other sources [37]. Previously, it was reported that increased interleukin-1α known as one of the SASPs accelerates skin pigmentation by enhancing tyrosinase activity [38]. Kim et al., showed that adipocyte-MSC-CM inhibited melanin synthesis and tyrosinase activity in mouse melanoma cells, while the expression level of MITF was not altered by adipocyte-MSC-CM [20]. In addition Hwang et al recently showed that treatment of CM derived from neural stem cells (NSC-CM) inhibited melanin production by activation of Wnt inhibitors [39].

To investigate the effective paracrine factors from hUCB-MSCs on anti-melanogenesis, we analyzed hUCB-MSC-CM using several biochemical approaches, including cytokine array (unpublished data). In accordance with the NSC-CM results, hUCB-MSCs also secreted various molecules related to the Wnt signaling pathway. In addition we found that transforming growth factor-β1 (TGFβ1) was highly produced in hUCB-MSCs. Since, TGFβ1 can inhibit melanogenesis through activation of the MAPK signaling pathway, we further evaluated the expression of MAPK proteins in cells-treated with hUCB-MSC-CM. As shown in Fig 4, we found that treatment of hUCB-MSC-CM induced ERK activation, which leads to proteasomal degradation of MITF in melanocytes. Moreover, treatment of an ERK inhibitor, PD98059, recovered the reduction of melanin contents by hUCB-MSC-CM in normal mouse melanocytes and in an artificial skin model (Figs 4 and 5). Expression of MITF can be regulated at the transcriptional level and via post-translational modification. The activated ERK stimulates phosphorylation of MITF on serine 73, eventually promoting its degradation via the ubiquitin-proteasome-dependent pathway [11, 30]. Treatment of hUCB-MSC-CM down-regulated the expression of MITF in melanocytes. Thus, additional investigation of TGFβ1-mediated signaling will help to further elucidate the underlying regulatory mechanisms of melanogenesis by hUCB-MSC-CM.

## Conclusion

In conclusion, we demonstrated that hUCB-MSC-CM significantly suppresses melanin synthesis via MITF degradation by ERK pathway activation. Although further molecular mechanism and clinical studies are needed, application of hUCB-MSC-CM shows great promise for cosmetic usages and to treat hyperpigmented skin disorders.
